# A new genus of the tribe Sarimini (Fulgoromorpha, Issidae) from the Guangxi Province of China

**DOI:** 10.3897/zookeys.912.39589

**Published:** 2020-02-17

**Authors:** Menglin Wang, Thierry Bourgoin

**Affiliations:** 1 Key Laboratory of Southwest China Wildlife Resources Conservation of the Ministry of Education, China West Normal University, Nanchong, Sichuan Province, 637009, China China West Normal University Nanchong China; 2 Institut de Systématique, Évolution, Biodiversité, ISYEB-UMR 7205 MNHN-CNRS-Sorbonne Université-EPHE, Muséum national d’Histoire naturelle, CP 50, 57 rue Cuvier, F-75005 Paris, France Sorbonne Université Paris France

**Keywords:** Fulgoroidea, molecular, morphology, new combination, new taxa, oriental, planthopper

## Abstract

A new genus with a new species *Eusarimissus hezhouensis***gen. nov. et sp. nov.** from Guangxi Province of China are described in the tribe Sarimini of the family Issidae. Molecular sequences of 18S, 28S and COXI genes are provided for the new taxon. Phylogenetic analysis places this taxon sister to a previously sequenced but not yet described Sarimini genus ‘*Eusarima* sp. 4’. Taxonomic notes are provided for the genus *Eusarima* Yang, 1994. The species Eusarima (Nepalius) iranica Gnezdilov & Mozaffarian, 2011 is transferred to the genus *Sarima* Melichar 1903.

## Introduction

Without obvious morphological apomorphy, the tribe Sarimini Wang, Zhang & Bourgoin, 2016 was only recently revealed after a molecular phylogenetic analysis of the planthopper family Issidae. It represents an important tribe which is sister to two other emblematic sister tribes in Hemisphaeriinae: the Parahiraciini Cheng & Yang, 1991 and the Hemisphaeriini Melichar, 1906 within the Issidae (Wang et al. 2016, Zhao et al. 2019). Currently, Sarimini includes 26 genera (Bourgoin 2020), plus several other genera already identified but not yet described which were provisionally labelled ‘Gen. nov.’, ‘*Eusarima* sp. 1’, ‘sp. 2’, and ‘sp. 4’ in Wang et al. (2016)’s analysis. The other taxa labelled with ‘Gen. nov. *apud Eusarima*’ and ‘*Eusarima* sp. 3’ were already described respectively as *Longieusarima* Wang, Zhang & Bourgoin, 2017 (Wang et al. 2017) and *Duplexissus* Wang, Zhang & Bourgoin, 2019 (Wang et al. 2019).

In this paper, we describe an additional new genus, representing a new species from Guangxi Province of China. We also provide taxonomic notes about the genus *Eusarima* Yang, 1994 from which one species, Eusarima (Nepalius) iranica Gnezdilov & Mozaffarian, 2011, is transferred to the genus *Sarima* Melichar, 1903. Finally, the number and the diversity of the taxa, which progressively joined Sarimini since its description, allow now a better understanding of its morphological characteristics.

## Materials and methods

The type specimens are deposited in China West Normal University, Nanchong, Sichuan Province, China. The specimens were collected by net capture during daytime. The genitalia were separated from the insect body using micro-scissors under a stereomicroscope Leica M205C, then transferred and boiled in a 5ml beaker with 10% NaOH solution for a few minutes until muscles were completely dissolved leaving only tegumentary structures. After rinsing in distilled water several times to clean the residual NaOH solution, genitalia were subsequently transferred to glycerine for final dissection and observation, and then stored under the specimen in a genitalia vial for final conservation. Photographs for external morphology and genitalia characters were taken using a Leica DFC camera attached to a Leica M205FA stereomicroscope and further refined with LAS X software. Morphological terminologies for male genitalia follow Bourgoin (1987), for female genitalia Bourgoin (1993), and for wing venation Bourgoin et al. (2015).

Total genomic DNA was extracted from the fore and middle legs of the holotype specimen using a Sangon Ezup column animal genomic DNA purification kit. The DNA of the genes (18S rRNA, 28S rRNA, COXI, Cytb) was amplified using the same primers and amplification procedures as in Wang et al. (2016). DNA sequencing was conducted by the Sangon Company (Shanghai, China). Contigs assembly was made using the software Seqman from package DNAstar v5.01 (www.dnastar.com). All sequences were registered in GenBank with accession numbers mentioned below.

MEGA v7.0 (Kumar et al. 2016) was used for performing alignments for a subset of taxa already analysed in Wang et al. (2016) but restricted to Sarimini plus the new genus. Phylogenetic analysis was performed using the software IQTREE v1.4.1 (Nguyen et al. 2015) with 10,000 bootstraps (Minh et al. 2013) and substitution models automatically selected with partitions unlinked. According to the results of Wang et al. (2016), genus *Darwallia* Gnezdilov, 2010 was chosen as an out-group for the analysis. FIGTREE v1.1.2 (Rambaut 2016) was used to visualize the tree.

## Taxonomy

### Hemisphaeriinae Melichar, 1906 (sec. Wang et al. 2016)

#### Sarimini Wang, Zhang & Bourgoin, 2016

##### Eusarimissus gen. nov.

Taxon classificationAnimaliaHemipteraIssidae

A3727780-F658-5CD3-A125-4E69C3BF3DF4

http://zoobank.org/16B6975F-3B6D-4EB7-BF14-3C7FDA91E48C

###### Type species.

*Eusarimissus hezhouensis* sp. nov., here designated.

###### Diagnosis.

**Figures 1–5.**
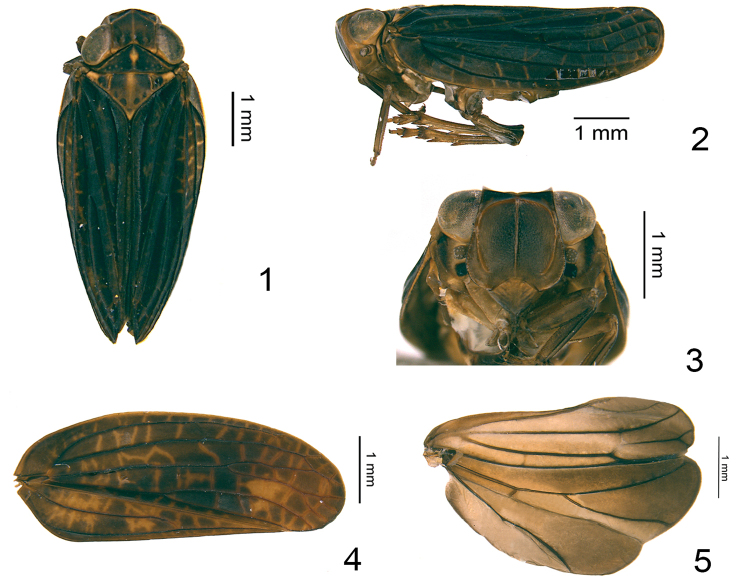
*Eusarimissus hezhouensis* sp. nov. **1** adult (holotype), dorsal view **2** adult (holotype), lateral view **3** adult (holotype), frontal view **4** forewing (paratype) **5** hind wing (paratype).

This new taxon appears similar to *Eusarima* but differs by: 1) Vertex much longer, around 1.3 times wider than long in midline (Fig. 1), but around 1.6 times wider in *Eusarima* (Chan and Yang 1994, fig. 45A); 2) MP vein forking late in apical 1/3, obviously after CuA (Fig. 4), while MP and CuA fork near middle, almost at the same level in *Eusarima* (Chan and Yang 1994, fig. 45C); 3) A2 lobe on hind wing as wide as Pcu-A1 lobe (Fig. 5), while larger in *Eusarima* (Chan and Yang 1994, fig. 45D); 4) dorsal lobe of periandrium without the posterolateral process (Fig. 9) in *Eusarima* (Chan and Yang 1994, fig. 45H).

From *Longieusarima*, *Eusarimissus* is easily separated by its shorter and wider vertex, the longer sublateral carinae of frons widely surpassing the level of the ventral margin of the antenna, and the general schema of the tegmina with a longer ScP+RA vein and a late-forking MP vein, well after the forking of CuA. Male genitalia also easily differentiated these two genera by the long subapical aedeagus processes in *Longieusarima*, shorter and in the apical 3/4 in *Eusarimissus*.

###### Description.

Head with compound eyes a little wider than pronotum and mesonotum (Fig. 1). Vertex hexagonal, a little wider than long in midline, median carina weakly present or absent on the disc; margins elevated, anterior margin obviously angularly convex at middle, lateral margins nearly straight and parallel, posterior margin angularly concave (Fig. 1). Frons rounded, wider than long, margins elevated; apical margin nearly straight, lateral margins expanding outward below antennae with lateral angles rounded (Fig. 3); median carina apparently elevated from apex extending to frontoclypeal suture (Fig. 3); sublateral carinae obviously elevated from the apex near to the base, but not reaching to the frontoclypeal suture, with lateral ventral angles rounded (Fig. 3). Frons smooth, without any tubercles (Fig. 3). Frontoclypeal suture strongly angularly convex (Fig. 3). Antenna with scape extremely short, pedicel rounded (Fig. 3). Clypeus smooth, without median carina (Fig. 3). Rostrum reaching to midcoxae, apical segment almost the same length with subapical one. Gena in lateral view oblique (Fig. 2). Pronotum triangular, almost same length with vertex in midline; margins elevated, anterior margin angularly protruded, posterior margin straight; median carina very weakly present or absent, with several inconspicuous tubercles on the disc (Fig. 1). Mesonotum triangular, a little wider than pronotum in midline, tricarinated on the disc, anterior margin straight (Fig. 1). Forewings obviously longer than broad, longitudinal veins distinctly elevated (Figs 1, 2, 4); costal area narrow, ScP+R forked at the base, ScP+RA and RP veins parallel, both extremely long, respectively extending to the apical 4/5 of costal margin and the apical margin (Figs 2, 4); MP straight, bifurcated into MP_1+2_ and MP_3+4_ at apical 1/3, forking again or not apically (Figs 2, 4); CuA bifurcated well before MP, slightly before Pcu and A1’s junction (Figs 1, 2, 4); clavus closed, CuP long, extending to the same level of ScP+RA, Pcu and A1 fused slightly beyond the middle of clavus (Fig. 4). Hind wings developed, of Sarimini type with 3-lobes (Fig. 5); Pcu-A1 lobe as wide as ScP-R-MP-Cu lobe, Pcu and A1 anastomosing at a medium distance, Pcu, A1_1_ and A1_2_ single (Fig. 5); A2 lobe developed, as wide as Pcu-A1 lobe, margin regularly slightly convex, A2 vein simple, non-branched (Fig. 5). Metatibia with two lateral spines on apical half (Fig. 2).

**Figures 6–11.**
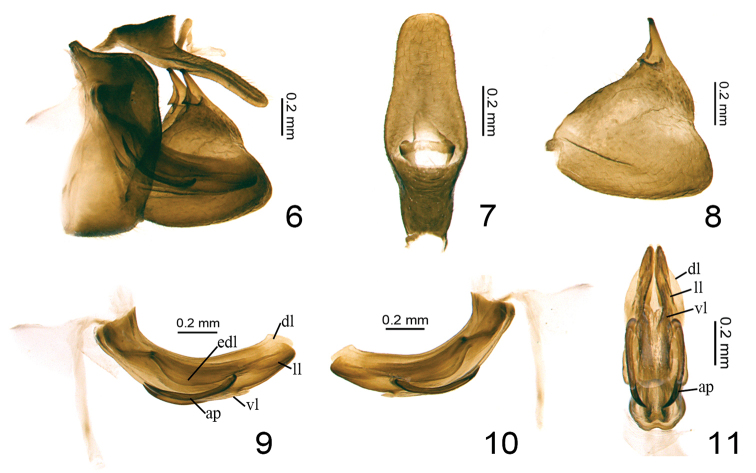
*Eusarimissus hezhouensis* sp. nov., holotype. **6** male genitalia, lateral view **7** male anal tube, dorsal view **8** gonostylus, lateral view **9** phallic complex, right lateral view **10** phallic complex, left lateral view **11** phallic complex, ventral view. Abbreviations: dl: dorsal lobe of periandrium; ll: lateral lobe of periandrium; vl: ventral lobe of periandrium; edl: expansion on dorsal lobe of periandrium; ap: aedeagus processes.

***Male genitalia.*** Anal tube in lateral view long and narrow, reaching to the posterior margin of gonostyli, basal part expanded, ventral margin nearly straight (Fig. 6). Pygofer in lateral view rectangular, obviously longer than broad, dorsal margin nearly straight, posterior margin slightly convex and parallel to anterior margin (Fig. 6). Gonostyli equilateral triangular in side view (Figs 6, 8). Capitulum of gonostylus short and spinous, with an auriform process near base (Figs 6, 8). Periandrium symmetrical (Figs 9, 10), with a dorsal lobe (dl), biforked lateral lobes (ll) and a ventral lobe (vl); dorsal and lateral lobes almost the same length, ventral lobe much shorter. Aedeagus with a pair of processes derived from the apical 3/4 (Figs 9, 10).

***Female genitalia.*** Gonoplacs in dorsal view fused at middle in basal 1/3 (Fig. 13); in lateral view long rectangular, longer than wide (Fig. 14). Gonapophysis IX in lateral view broad, dorsal margin sinuate, basal 1/3 with a needle-like process (Fig. 15); ventral margin concave (Fig. 15). Gonapophysis IX in dorsal view nearly triangular, the basal half broader than the apical half (Fig. 16). Gonospiculum bridge in lateral view rectangular with a short needle-like process at base (Fig. 15). Anterior connective lamina of gonapophysis VIII subtriangular, with teeth at apex and teeth on the outer lateral margin, inner lateral margin without teeth (Fig. 18). Endogonocoxal process membranous (Fig. 18). Gonocoxa VIII long rectangular, posterior and anterior margins parallel and concave (Fig. 18).

**Figures 12–18.**
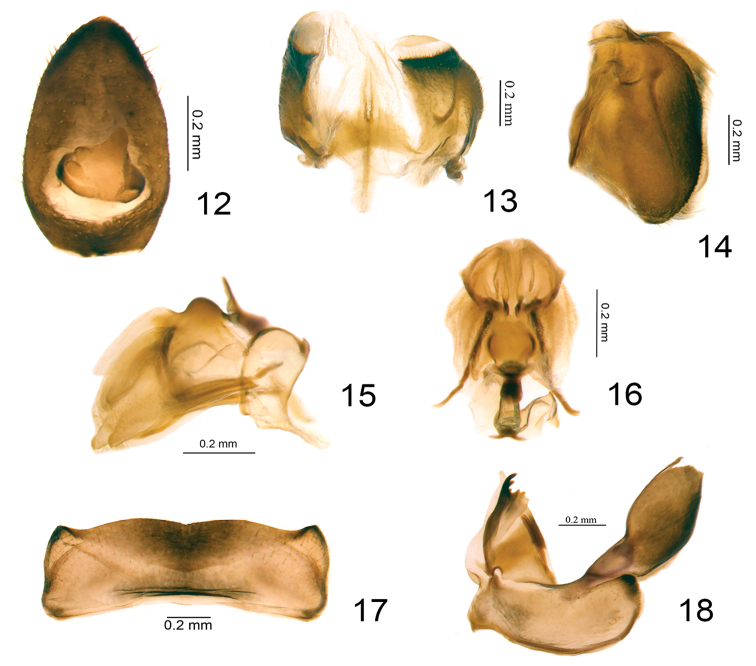
*Eusarimissus hezhouensis* sp. nov., paratype. **12** female anal tube, dorsal view **13** gonoplacs, dorsal view **14** gonoplacs, lateral view **15** gonapophysis IX and gonospiculum bridge, lateral view **16** gonapophysis IX and gonospiculum bridge, dorsal view **17** sternite VII, ventral view **18** gonocoxa VIII and gonapophysis VIII, ventral view.

###### Etymology.

This name is an arbitrary association between the genera names “*Eusarima*” and “*Issus*” referring to the close relationship of this genus to *Eusarima* in the Issidae tribe Sarimini. The gender is masculine.

###### Distribution.

China (Guangxi Province).

##### Eusarimissus hezhouensis sp. nov.

Taxon classificationAnimaliaHemipteraIssidae

9EF96229-89AA-52E5-81A4-3AEA7EC2DC3B

http://zoobank.org/41895156-B94F-48B1-A7FC-E82FEFD598F1

###### Diagnosis.

This new species looks similar to the species Eusarima (Eusarima) triphylla (Che, Zhang & Wang, 2012) known also from Guangxi Province, but it differs by: 1) the early bifurcation of CuA before MP (Fig. 4), while almost at the same level in E. (E.) triphylla (Che et al. 2012, figs 3, 6); 2) the male anal tube widest slightly below middle in dorsal view (Fig. 7), while widest in apical 1/2 in the latter species (Che et al. 2012, fig. 9); 3) the female anal tube shorter, 1.6 times longer in the length at middle than widest part in dorsal view (Fig. 12), while 2.4 times in E. (E.) triphylla (Che et al. 2012, fig. 13); 4) the apical margin of female sternite VII shallowly concave (Fig. 17), while roundly convex medially in E. (E.) triphylla (Che et al. 2012, fig. 14); 5) the single Pcu of the hind wing, while triforked in E. (E.) triphylla (Che et al. 2012, fig. 7).

###### Type materials.

***Holotype***: ♂, China: Guangxi Province, Hezhou, Qichong Nature Reserve, 24°13'6"N, 110°48'34"E, 180 m, 7.viii.2018, coll. Feilong Yang & Kun Zhao. ***Paratypes***: 2♂♂, 1♀, same data as holotype.

###### Description.

***Length***: male (including forewings) (*N* = 3): 6.1–6.3 mm; female (including forewings) (*N* = 1): 6.4 mm.

***Coloration*.** Vertex almost dark brown, the midline slightly yellow; anterior margin yellow; posterior margin yellow with some black (Fig. 1). Compound eyes silvery white (Figs 1–3). Frons brown, anterior area from apical margin to the middle level of compound eyes darker; median and sublateral carinae tawny (Fig. 3). Antennae brown (Fig. 3). Clypeus dark brown with some yellow (Fig. 3). Rostrum tawny. Gena tawny (Fig. 2). Pronotum dark brown, with yellow midline, some specimens with a yellow ovate marking at middle, the disc scattered with 6–8 yellow tiny nodules on each side; anterior margin yellow, posterior margin black (Fig. 1). Mesonotum mostly dark brown, midline broad yellow, sublateral carinae yellow; the basal median area with four large black spots, the triangular intersection of the anterior and posterior margins yellow (Fig. 1). Forewings dark brown, longitudinal veins black, with irregular pale-yellow transverse veins (Figs 1, 2, 4). Hind wing pale brown, darker apically (Fig. 5). Legs all tawny (Figs 2, 3).

***Head and thorax*.** Vertex 1.3 times wider than long in midline, posterior margin with the protruded level little shallower than anterior margin (Fig. 1). Frons 0.8 times longer in middle than broad at widest part, 1.4 times wider at the widest part than apical margin (Fig. 3); sublateral carinae obviously elevated from apex extending to basal 1/6, not reaching the clypeus (Fig. 3). Pronotum with posterior margin 2.6 times wider than long in midline (Fig. 1). Mesonotum with anterior margin 1.7 times wider than long in midline (Fig. 1). Forewings 2.6 times longer in longest part than widest part, MP vein firstly forked at apical 1/3, MP_1+2_ forked again at apical 1/5 (Fig. 2) or unforked (Fig. 4), MP_3+4_ bifurcate at apical 1/6 (Fig. 4) or unforked (Fig. 2); CuA forked near middle, before the first fork of MP, CuA_1_ simple and sinuate, CuA_2_ simple and straight (Figs 2, 4). Metatibiotarsal formula: 2–6/8/2.

***Male genitalia*.** Anal tube slender in lateral view, broad in basal 1/3 then gradually narrowing to the apex (Fig. 6); in dorsal view anal tube long cylindrical, broadest below middle, the length in midline 2.7 times longer than the widest part, dorsal margin almost straight (Fig. 7); anal opening located below middle (Fig. 7); epiproct exceeding to the middle of anal tube (Fig. 7). Pygofer with the highest length in midline 2.0 times longer than the width at middle (Fig. 6); dorso-apical angle obtuse and oblique (Fig. 6). Gonostylus in lateral view with dorsal margin oblique and almost straight, posterior margin slightly concaved at middle, ventral margin deeply convex in the apex with caudo-ventral angle rounded (Figs 6, 8). Capitulum of gonostylus spiniform, with a small auriform process near base (Figs 6, 8). Periandrium tubular, dorsal lobe with ventral margin expanded (edl) near middle, fused with lateral lobes at basal 1/3 (Figs 9, 10); in ventral view dorsal lobe slightly broaden near the apex, lateral lobes bifurcate at middle near the apex, ventral lobe with dorsal margin slightly concave at middle (Fig. 11); ventral lobe (vl) only reaching the apical 1/3 of periandrium. Aedeagus symmetrical, with a pair of hooked processes (ap) derived from apical 1/3 extending along the ventral margin of periandrium reaching to the basal 1/3 (Figs 9, 10), in ventral view this pair of processes slightly curved inward (Fig. 11).

***Female genitalia*.** Anal tube in dorsal view conical, 1.6 times longer in midline than widest part (Fig. 12); apical margin sharp, lateral margins gradually broadening from apex to basal 1/3 (Fig. 12); anal opening situated at basal 1/3 (Fig. 12). Gonoplacs in dorsal view with outer lateral margins roundly convex, the apical part and median part membranous (Fig. 13); in lateral view rectangular, 1. 6 times longer in longest part than widest part, the apical margin rounded (Fig. 14). Gonapophysis IX in lateral view broad, dorsal margin elevated and convex at middle, basal 1/3 with a needle-like process (Fig. 15). Gonapophysis IX in dorsal view widest near middle, basal half broader than apical half, outer area concave inward near apical 1/3 (Fig. 16). Anterior connective lamina of gonapophysis VIII with three teeth at apex and three teeth on the outer lateral margin, inner lateral margin without teeth (Fig. 18). Endogonocoxal process reaching to the same level of apex of anterior connective lamina (Fig. 18). Gonocoxa VIII long rectangular, perpendicular the gonapophysis VIII (Fig. 18). Apical margin of sternite VII mostly straight, with middle part very shallowly incised and two prominent dorso-lateral angles in ventral view (Fig. 17).

###### Etymology.

The name refers to the type locality of the species.

###### Phylogeny.

**Figure 19.**
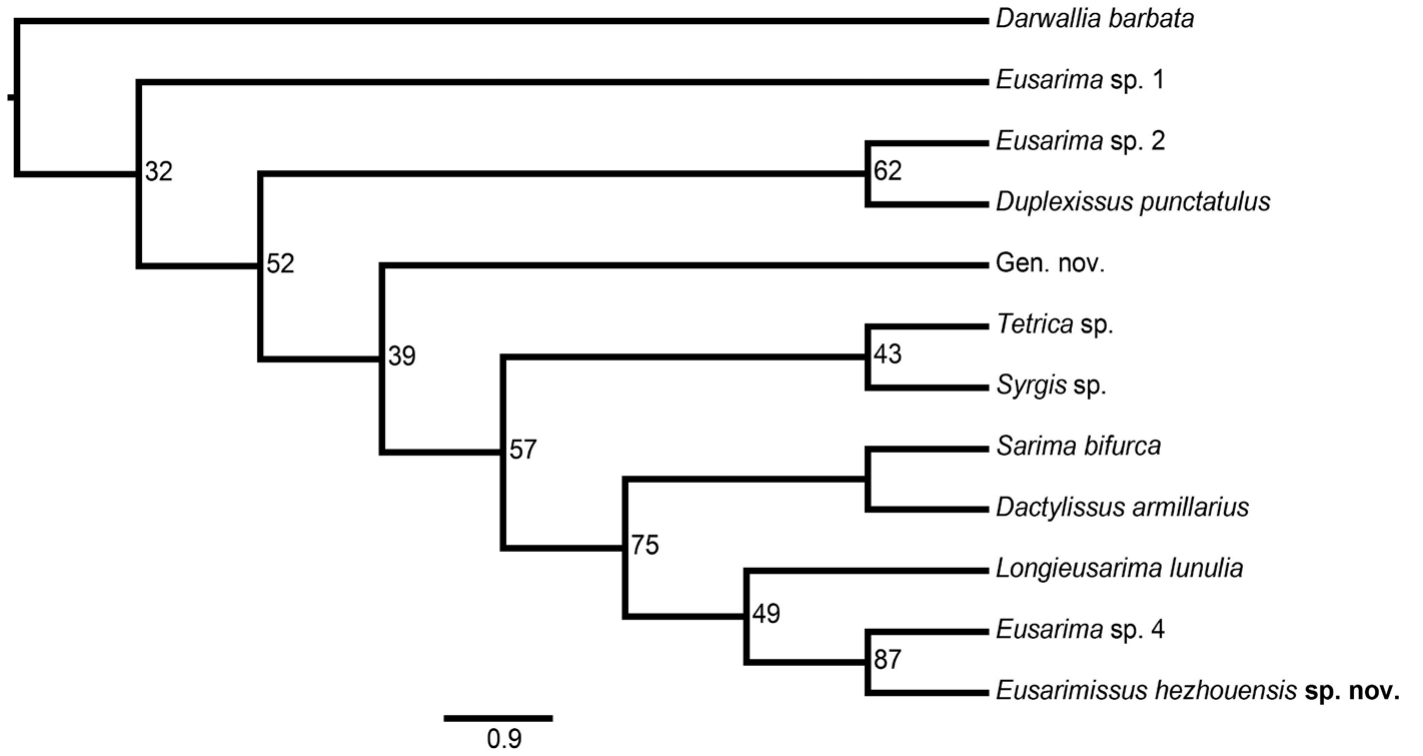
Maximum likelihood tree of Sarimini species based on combined sequences (18S, 28S, COXI, Cytb) with *Darwallia* as outgroup. At each node, values denote ML bootstrap support. The name ‘Gen. nov.’, ‘*Eusarima* sp. 1’, ‘sp. 2’, and ‘sp. 4’ refer to the same taxa as in Wang et al. (2016).

The molecular sequences obtained were registered in GenBank with the following accession numbers: MN955873 (18S, primers: 3F–Bi + A2–9R), MN955872 (28S D3–D5, primers: Ai–D4D5r), MN955852 (28S D6–D7, primers: EE–MM), MN954323 (COXI). Cytb sequence was failed to obtain. Molecular analysis based on available sequences of the 18S rRNA, 28S rRNA, COXI and Cytb genes confirms the morphological data positioning the new taxon in Sarimini. The species takes place as sister to a non-described but already sequenced Sarimini species *Eusarima* sp. 4 in Wang et al. (2016), both being sister taxa to *Longieusarima lunulia* Wang, Bourgoin & Zhang, 2017 (Fig. 19). Barcoding part of COXI gene of *Eusarimissus hezhouensis* sp. nov. differs by 107 bp and 115 bp from *Eusarima* sp. 4 and *L. lunulia* Wang, Bourgoin & Zhang, 2017 respectively over the total length of 681 bp.

## Discussion

Analysis of all Sarimini genera currently available for a molecular phylogeny, places *Eusarimissus* gen. nov. as sister to *Eusarima* sp. 4 which are both sister to the genus *Longieusarima*. Morphologically, *Eusarimissus* gen. nov. resembles the genus *Eusarima* from which it could be easily separated by the forewing venation and the presence of the posterolateral processes of the dorsal lobe of the periandrium in the latter genus.

*Eusarima* is a large genus including 37 species (Bourgoin 2020). It was divided into two subgenera: *Eusarima* and *Nepalius* Dlabola, 1997, the latter synonymized with the former by Gnezdilov (2009) before being revalidated as a subgenus by Gnezdilov and Mozaffarian (2011). Subgenus Nepalius currently contains three species distributed in the Western Palaearctic area (Nepal, Iran and Pakistan). It represents probably a separated valid independent genus with its type species Eusarima (Nepalius) helleriana (Dlabola, 1997) and Eusarima (Nepalius) albifrons Gnezdilov, 2016. However, according to its forewing conformation (particularly by its short recurved ScP+RA to RP), Eusarima (Nepalius) iranica Gnezdilov & Mozaffarian, 2011 is here transferred in the genus *Sarima* Melichar, 1903 as *Sarima iranica* (Gnezdilov & Mozaffarian, 2011) comb. nov. Because *Sarima* is probably also a composite genus in need of revision, we don’t exclude the possibility that *S. iranica* could belong to an independent genus itself. Thirty-one other species in the nominal subgenus Eusarima occur in Taiwan, which is regarded as an example of extensive insular diversification (Gnezdilov 2016). Two more species were also described from Japan and another one from Guangxi Province, China. The latter, Eusarima (Eusarima) triphylla, differs from all other *Eusarima* and Sarimini species by several characters that need to be confirmed: a CuA_1_ vein apically single, a 3-forked Pcu vein and an incomplete A1 vein. Because the published figure looks rather schematic (Che et al. 2012, fig. 7), this venation needs to be rechecked for confirmation and sequencing this species for comparison with other Sarimini taxa would be of great interest as obviously its generic placement remains dubious. Unfortunately, we don’t also know the precise phylogenetic placement of the genus *Eusarima* itself within the Sarimini: all species analysed in Wang et al. (2016) and labelled ‘*Eusarima* sp.’, although quite close to *Eusarima* species, are not true *Eusarima* taxa.

The diversity of the taxa that progressively joined the Sarimini tribe, allows us now to better clarify the morphological characteristics of the group. Indeed, within the Issidae, Sarimini shares a specific 3-lobed hind wing conformation with an A2 lobe as wide or wider than the other lobes, often notched at the A2 extremity, and with several venation characteristics that seems emerging as a specific combination for the group: lobes with non-reticulated venation, Pcu-A1 lobe usually without transverse veins, cubital band area between CuP and Pcu always much wider than the intra-cubital band area between CuA and CuP, MP single, Pcu anastomosing at some distance with A1 anterior branch, Pcu, A1_1_, A1_2_ and A2 single.

## Supplementary Material

XML Treatment for Eusarimissus

XML Treatment for Eusarimissus hezhouensis
